# Adolescent Mental Health, Contraceptive Knowledge, and Teen Pregnancy Risk: A Systematic Review

**DOI:** 10.3390/healthcare13212660

**Published:** 2025-10-22

**Authors:** Denisa Hinoveanu, Ileana Enatescu, Catalin Dumitru, Patricia Octavia Mazilu, Daniel Popa, Cristina Anemari Popa, Mihail-Alexandru Badea, Felicia Marc, Adrian Gluhovschi

**Affiliations:** 1 Department of Obstetrics and Gynecology, “Victor Babes” University of Medicine and Pharmacy Timisoara, 300041 Timisoara, Romania; adriana.hinoveanu@umft.ro (D.H.); dumitru.catalin@umft.ro (C.D.); gluhovschi.adrian@umft.ro (A.G.); 2 Doctoral School, “Victor Babes” University of Medicine and Pharmacy, Eftimie Murgu Square 2, 300041 Timisoara, Romania; 3 Discipline of Neonatology, Victor Babes University of Medicine and Pharmacy, 300041 Timisoara, Romania; enatescu.ileana@umft.ro; 4 Faculty of Medicine, “Victor Babes” University of Medicine and Pharmacy Timisoara, Eftimie Murgu Square 2, 300041 Timisoara, Romania; patrimazilu@yahoo.com; 5 Department of Medical Rehabilitation, Faculty of Medicine, “Victor Babes” University of Medicine and Pharmacy Timisoara, Eftimie Murgu Square 2, 300041 Timisoara, Romania; popa.daniel@umft.ro; 6 Department of Genetics, Genomic Medicine Centre, Faculty of Medicine, “Victor Babes” University of Medicine and Pharmacy Timisoara, Eftimie Murgu Square 2, 300041 Timisoara, Romania; popa.cristina@umft.ro; 7 Oncology-Hematology Research Unit, Romanian Academy of Medical Sciences, “Louis Turcanu” Timisoara European Hemophilia Treatment Centre, 300011 Timisoara, Romania; 8 Dermatology Department, The George Emil Palade University of Medicine, Pharmacy, Science, and Technology, 540139 Targu Mureș, Romania; 9 Department of Medical Sciences, Faculty of Medicine and Pharmacy, University of Oradea, 410073 Oradea, Romania

**Keywords:** adolescent, depression, anxiety, contraceptive knowledge, misinformation, delayed contraception, teenage pregnancy

## Abstract

**Background**: Adolescent depressive and anxiety symptoms may erode motivation and problem-solving needed for timely contraception, while online information quality is uneven. We synthesized evidence linking mental health, contraceptive knowledge/access, and teen pregnancy risk. **Methods**: Following PRISMA-2020, we searched PubMed, Embase, and Scopus to 7 July 2025 for primary studies including adolescents that measured validated mental health symptoms or psychiatric settings and reported contraceptive knowledge/access/behavior and/or teen pregnancy outcomes. Two reviewers screened/extracted data; risk of bias was appraised with the Newcastle–Ottawa Scale and ROBINS-I. Given heterogeneity, we conducted narrative synthesis. **Results**: Six U.S.-based studies met the criteria, spanning community colleges, a national cohort, school surveillance, psychiatric inpatient care, and pediatric emergency departments (samples: n = 143 to weighted N = 29,755). Depressive symptoms were associated with contraception access (adjusted odds ratio [aOR] 1.58, 95% CI 1.27–1.96) and anxiety/stress with similar risk (aOR 1.46, 1.17–1.82). A first depressive episode in the same year as sexual debut increased teenage pregnancy hazard (adjusted hazard ratio 2.70, 1.15–6.34). School surveillance showed mental health indicators correlated with contraception non-use at last sex (odds ratios 1.78–2.71). Among psychiatric inpatients, not knowing where to obtain contraception and access difficulties strongly predicted interest in information (aOR 2.96–3.33) and initiation (aOR 2.85–4.72). In a pediatric emergency department trial, same-day initiation occurred in 26.8% versus 3.1% under usual care. **Conclusions**: Evidence directly linking adolescent mental health symptoms to teen pregnancy is limited (one study), whereas multiple studies show associations with contraception knowledge/access and delayed or non-use, suggesting plausible indirect pathways to pregnancy risk. These findings support hypothesis-generating, integrated approaches and highlight the need for studies with teen pregnancy endpoints.

## 1. Introduction

Adolescent mental health concerns remain strikingly prevalent in the post-social-media era. In the nationally representative 2023 Youth Risk Behavior Survey (YRBS), 39.7% of high school students reported persistent sadness/hopelessness, 20.4% seriously considered suicide, and 9.5% attempted suicide, burdens that vary by sex, race/ethnicity, and sexual identity [1]. The 2023 YRBS methods update underscores how these indicators are measured and interpreted across subgroups [2]. At the same time, U.S. teen birth rates have continued their long decline, but remain uneven across populations, sharpening the policy imperative to sustain prevention while addressing new barriers to timely contraception [3]. Globally, adolescent pregnancy remains a public health priority, with higher burdens reported in many low- and middle-income countries. Information environments (school-based curricula, family/community norms, and digital media) and service pathways vary widely across settings.

Adolescents increasingly encounter contraception content online before or instead of clinical counseling [4,5]. The quality of these messages is mixed and often difficult to appraise, which can shape beliefs about methods, side effects, and safety. In this context, measuring contraception knowledge and access alongside mental health status is important because information gaps or misperceptions may interact with affective symptoms to influence timing and consistency of use [6]. A 2025 systematic appraisal of top contraception-related social media content further documents widespread misinformation and a tilt toward “natural” methods without appropriate risk disclosures [7]. Together, these studies point to a powerful information environment in which adolescents encounter compelling and uneven contraception guidance before or instead of clinical counseling. In the U.S., classroom-based sex education is variably implemented across states and districts, with differences in scope, mandating, and emphasis; adolescents therefore often encounter a mixed ecosystem of school curricula and online sources when forming contraception beliefs [8].

Clinical guidance has long emphasized confidential, youth-friendly, and method-neutral contraceptive care. The American Academy of Pediatrics (AAP) policy, updated in 2025 and building on its 2014 statement, recommends developmentally appropriate counseling, ready access to highly effective methods, and equity-informed practices [9,10]. The American College of Obstetricians and Gynecologists (ACOG) likewise endorses long-acting reversible contraception (LARC) as safe, acceptable, and highly effective for adolescents who choose these methods [11]. Yet these recommendations assume intact help-seeking capacity; depressive and anxiety symptoms, plus confusing online content, can erode motivation, navigation, and follow-through precisely when contraception decisions are time-sensitive.

Empirical studies link mental health symptoms to contraceptive behaviors associated with higher pregnancy risk. In prospective work, depressive and stress symptoms predicted weeks of non-use or use of less-effective methods among young women [12], and depressive symptoms were associated with less consistent use over time [13]. Among depressed adolescent and young adult females, condom errors and problems were more common, highlighting cognitive/behavioral pathways by which symptoms can undermine protection [14]. These findings suggest that even modest elevations in distress may disrupt the planning, persistence, and problem-solving that contraception often requires.

Timing may amplify risk. In a national cohort, having a first depressive episode in the same year as sexual debut was associated with markedly higher hazards of teenage pregnancy and sexually transmitted infection, consistent with a sensitive window when symptoms, skills, and supports jointly shape early sexual health trajectories [15]. Population surveillance likewise shows that mental health burdens frequently cluster with other risk exposures—e.g., adverse childhood experiences (ACEs)—that correlate with sexual risk behaviors and poorer outcomes [16]. These patterns strengthen the rationale for pairing mental health support with concrete contraception knowledge and access at the earliest stages of sexual activity.

Promising delivery points exist. In pediatric emergency departments (EDs), randomized and mixed-methods trials demonstrate the feasibility and acceptability of counseling with same-day initiation, with many adolescents starting a method during the visit and clinicians rating workflows as workable [17,18]. Earlier ED interventions showed meaningful initiation but also exposed barriers to follow-up and access logistics that must be solved for sustained use [19]. In psychiatric inpatient settings, adolescents frequently report not knowing where to obtain contraception or difficulty accessing it—and these knowledge/access gaps strongly correlate with immediate interest in receiving contraception, pointing to high-yield opportunities during mental health care encounters [20].

Therefore, the objective of the current study was to synthesize primary studies linking (1) adolescent mental health symptoms or psychiatric settings with (2) contraception knowledge/access and/or contraceptive behaviors, and (3) teen pregnancy outcomes, where available.

## 2. Materials and Methods

### 2.1. Protocol and Registration

We followed PRISMA 2020 reporting guidance [21]. The review was registered on OSF (osf.io/jxdzc) after initial screening (retrospective registration); eligibility criteria and analysis plans were specified a priori and were not altered thereafter. The review question (PICO) targeted Population: adolescents and emerging adults (primarily 14–19; extended to 24–29 if adolescent subgroup effects or direct teen-relevant outcomes were reported); Exposure: validated depressive/anxiety/stress measures or psychiatric settings; Comparator: asymptomatic peers or lower-symptom strata; Outcomes: (a) contraceptive knowledge/access (including “not knowing where to get contraception,” difficulty accessing care, or documented misinformation exposure), (b) contraceptive behavior (initiation, delays, non-use/inconsistent use), and/or (c) pregnancy outcomes (teen pregnancy incidence or closely linked risk markers). We did not prospectively register the protocol; however, we a priori specified eligibility, data items, and analysis plans, including risk of bias appraisal (NOS/ROBINS-I as applicable) and a narrative synthesis strategy due to anticipated heterogeneity in mental health instruments and knowledge measures.

### 2.2. Eligibility Criteria

Inclusion: (i) Primary research; (ii) sample includes adolescents (or emerging adults with adolescent-relevant subgrouping); (iii) mental health exposure via validated instrument or recruitment from a psychiatric setting; (iv) at least one contraceptive knowledge/access/behavior variable or a teen pregnancy outcome; (v) English; (vi) PubMed, Embase, and Scopus-indexed; (vii) sufficient quantitative information to extract (prevalence/effect estimates) or clearly report as NR where unavailable. Where studies enrolled mixed age ranges, adolescent-specific endpoints (including teen pregnancy) were required for inclusion; this criterion yielded one eligible teen pregnancy study during the review window. Exclusion: Narrative/editorial pieces without primary data; studies of postpartum/postabortion adults only; outcomes limited to STI knowledge without contraception; purely values/preferences without knowledge/access; systematic reviews (used for context only). Adolescents were defined per original studies; where studies enrolled 18–29-year-olds, we retained them if they explicitly analyzed adolescents or reported teen-relevant access/behavior outcomes.

We anticipated variability across settings (school surveys, community colleges, ED/psychiatric units) and accepted designs accordingly. Social media misinformation studies were included only if they connected to contraceptive knowledge/behavior or teen outcomes among adolescents; broader misinformation/mental health papers informed the background only.

### 2.3. Search Strategy

We searched PubMed, Embase, and Scopus databases from inception to 7 July 2025 using controlled vocabulary and free-text terms combining four concepts: (adolescents) AND (depression OR anxiety OR stress OR mental health) AND (contraception OR contraceptive knowledge OR access OR delay OR initiation) AND (pregnancy OR teen pregnancy), plus terms for misinformation/TikTok/social media. We complemented sensitive strings with targeted lookups for known high-yield papers in adolescent emergency department and psychiatric settings. Representative queries are in the citations and include the community college mental health/delay study; depression timing and teen pregnancy; psychiatric inpatient contraception knowledge; ED contraception initiation feasibility; school-based YRBS analysis; and TikTok/contraceptive misinformation for context. We limited to English and human subjects. Reference lists of included studies and key commentaries were hand-searched.

PubMed (searched through 7 July 2025): We used the following Boolean combination with MeSH and free-text terms, applying Human and English filters for 1990–2025: (‘Adolescent’[Mesh] OR adolescent* OR teen* OR youth) AND (depress* OR anxiety OR ‘Stress, Psychological’[Mesh] OR mental health OR mental health) AND (contracept* OR ‘Contraception’[Mesh] OR birth control OR family planning OR LARC) AND (knowledge OR misconception* OR misinformation OR access OR delay* OR initiation OR ‘patient acceptance of health care’[Mesh]) AND (‘Pregnancy in Adolescence’[Mesh] OR teen pregnan* OR adolescent pregnan*).

Embase via Ovid (searched through 7 July 2025): We limited the search to Human, English, 1990–2025, with the following fields: (adolescent* OR teen* OR youth) AND (depress* OR anxiety OR mental health OR psychological stress) AND (contracept* OR birth control OR family planning OR LARC) AND (knowledge OR misconception* OR misinformation OR access OR delay* OR initiation) AND (adolescent pregnancy OR teen pregnancy).

Scopus (searched through 7 July 2025): We limited the search to English and years 1990–2025, using TITLE-ABS-KEY fields: (adolescent* OR teen* OR youth) AND (depress* OR anxiety OR ‘mental health’ OR ‘psychological stress’) AND (contracept* OR ‘birth control’ OR ‘family planning’ OR LARC) AND (knowledge OR misconception* OR misinformation OR access OR delay* OR initiation) AND (‘teen pregnancy’ OR ‘adolescent pregnancy’).

### 2.4. Study Selection and Data Extraction

**Figure 1:**
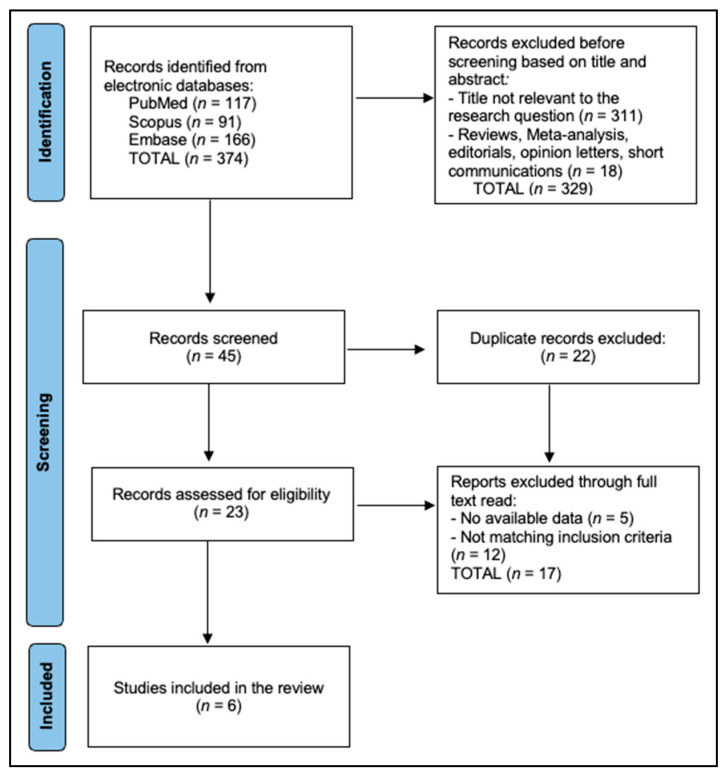
PRISMA Flowchart.

Two reviewers independently screened titles/abstracts, then full texts in duplicate. Disagreements were resolved by consensus. We extracted country, setting, design, sample size/age span, mental health measure (instrument, cut-points), knowledge/access variables (e.g., “not knowing where to get contraception,” difficulty accessing services, delay), behavior outcomes (initiation, non-use/inconsistent use), and teen pregnancy outcomes (incidence, hazards). We captured effect sizes (aOR, aHR, β) with 95% CIs and adjusted covariates; when effects were not reported or not estimable, we recorded NR. Where studies provided prevalence without multivariable associations (e.g., YRBS domain counts), we extracted those. For psychiatric/ED settings, we captured proportions interested in initiation after counseling. For mixed age studies, we extracted adolescent-specific estimates only; older young adult data were excluded from tables and synthesis. We prespecified to avoid meta-analysis unless ≥3 studies provided commensurate measures for the same outcome (criteria not met). We did not contact authors for additional or missing data; where adjusted estimates were not reported in accessible sections, these are recorded as not reported (NR) and interpreted cautiously. Study inclusion flowchart is presented in Figure 1.

### 2.5. Risk of Bias

We used the Newcastle–Ottawa Scale [22] for observational cohorts and cross-sectional designs (modified for exposure ascertainment quality and representativeness), ROBINS-I for quasi-experimental/ED interventions, and qualitative appraisal for interview-based components nested in trials (informative but not pooled). We summarized certainty narratively, noting when associations might reflect residual confounding (e.g., socioeconomic status, relationship violence) or selection (clinical vs. community samples). Given instrument diversity (CES-D, DASS-21, single-item YRBS mental health indicators) and outcome heterogeneity (delayed contraception vs. initiation vs. teen pregnancy), we synthesized direction and magnitude narratively and emphasized load-bearing estimates that were adjusted and time-anchored (aORs and aHRs), as presented in Table 1.

## 3. Results

Of the six studies, one reported teen pregnancy outcomes directly; the remainder reported contraception knowledge/access or use behaviors linked to pregnancy risk. All six included studies were conducted in the United States and span community colleges, national surveillance, school-based surveys, psychiatric inpatient care, and pediatric emergency departments, with sample sizes from 143 to a weighted N = 29,755 and ages primarily in adolescence (13–18) with one older adolescent/young adult cohort (18–29) [15,19,20,23,24,25]. Harper et al. enrolled 1665 students across 29 sites in Texas and California and measured depressive symptoms (CES-D) plus anxiety/stress (DASS-21) in relation to delayed contraception [23]; Vafai et al. analyzed 1025 sexually active girls (13–18) in the NCS-A using CIDI-based DSM-IV to time the first depressive episode relative to sexual debut and subsequent teenage pregnancy [15]; Casola et al. summarized 2015 Philadelphia YRBS data (weighted N = 29,755; sexually active subset) using past-year mental health indicators (sad/hopeless ≥ 2 weeks, suicidal ideation, suicide attempt) and contraception non-use at last sex [24]; Berlan et al. studied 451 psychiatric inpatients (14–17) and captured contraception knowledge/access gaps (e.g., “did not know where to get contraception,” “difficulty getting to access”) and interest in information/initiation during admission with adjusted models [20]; Hoehn et al. conducted a pilot randomized trial (n = 143) testing ED-based counseling/offers of same-day contraception and measured same-day initiation at the index visit [19]; Mollen et al. provided an implementation perspective on leveraging the pediatric ED for access (narrative outcomes) [25], as presented in Table 2.

Objective prevalences show high burdens at multiple points along the pathway. In community colleges, 35% of older adolescents/young adults reported delaying needed contraception (May 2020–March 2023 across 29 sites) [23]. In the national NCS-A cohort, 13% experienced a first teenage pregnancy during observed risk time after sexual debut [15]. In the 2015 Philadelphia YRBS sexually active subset, contraception non-use at last sex was 33%, with mental health indicators common (sad/hopeless 34%, suicidal ideation 13%, suicide attempt 11%) [24]. In psychiatric inpatients, 51.3% were interested in contraception information and 21.4% in initiation during hospitalization [20]. In the pediatric ED randomized pilot, same-day initiation occurred in 26.8% of adolescents in the intervention arm versus 3.1% in usual care (*p* = 0.001) at the index visit [19]. The ED implementation overview summarized programmatic feasibility without new quantitative endpoints [25], as seen in Table 3.

**Figure 2:**
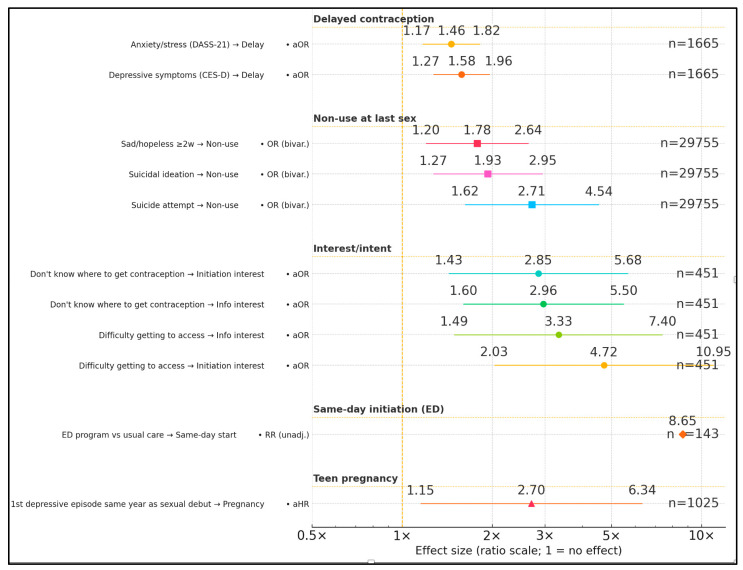
Forest plot of factors that influence mental health.

Across studies, mental health symptoms and access/knowledge gaps were consistently associated with riskier contraceptive outcomes. In Harper et al., depressive symptoms predicted delayed contraception and anxiety/stress showed a similar association, with adolescents at higher risk than young adults [23]. In Vafai et al., having the first depressive episode in the same year as sexual debut doubled-to-tripled the hazard of teenage pregnancy [15]. In Philadelphia YRBS data (bivariate, weighted), suicidal ideation, sadness/hopelessness, and suicide attempt were linked to contraception non-use; final adjusted estimates were not reported in accessible sections [24]. In psychiatric inpatients, not knowing where to obtain contraception increased interest in information and initiation, while difficulty accessing care had even larger effects [20]. The ED randomized pilot showed a large absolute difference in same-day initiation (26.8% vs. 3.1%), though an adjusted effect size was not reported [19], and the ED perspective provided qualitative feasibility insights without quantitative associations [25], as described in Table 4 and Figure 2.

## 4. Discussion

### 4.1. Summary of Evidence

Across heterogeneous observational designs, we observed consistent associations between adolescent mental health symptoms, contraception knowledge/access barriers, and delayed or non-use; these findings are hypothesis-generating rather than causal. Across community, clinical, and nationally representative samples, depressive, anxiety, and stress symptoms were repeatedly linked to delays in obtaining contraception, inconsistent or non-use of contraceptives, and elevated risk for teenage pregnancy. Importantly, the magnitude of these associations—such as the nearly threefold hazard of teenage pregnancy when a first depressive episode coincided with sexual debut—suggests that the interplay between mental health and contraceptive behavior is both temporally and clinically significant. This pattern persisted across diverse settings, from high schools to psychiatric inpatient units, indicating a pervasive, cross-contextual vulnerability that warrants integrated prevention strategies.

A notable contribution of this review is the identification of knowledge and access deficits as high-yield intervention points, particularly among adolescents in psychiatric care or emergency department (ED) settings. Studies demonstrated that adolescents who did not know where to obtain contraception or who faced logistical barriers were significantly more likely to express interest in information and initiation during care encounters. The ED-based intervention trial further demonstrated that same-day initiation is feasible, with a more than an eightfold absolute increase in uptake compared to usual care. These results suggest that high-contact points in mental health and acute care systems may serve as effective, underutilized venues for delivering contraceptive education and initiation—especially when paired with tailored counseling for adolescents experiencing psychological distress.

Here we situate our results within the broader literature and highlight where they converge and where they extend current evidence. First, the observed link between depressive/anxiety symptoms and riskier contraceptive outcomes is consistent with longitudinal and cross-sectional data showing that adolescents with internalizing symptoms initiate sex earlier, report more unprotected sex, and cluster multiple risks. In a U.S. school-based cohort, depressive symptoms prospectively predicted greater sexual risk behaviors over time [26], and earlier work suggested that mood symptoms often precede (rather than follow) sexual debut and substance use [27]. Event-level and survey analyses similarly connect depressive affect to inconsistent condom use and other vulnerabilities [28]. Population studies from outside the U.S. reinforce this comorbidity, with depression tracking more sexual health problems and service need [29]. These patterns align with our synthesis: even modest elevations in distress appear sufficient to disrupt the planning and persistence that contraception requires, particularly in adolescence when executive control and care-navigation skills are still maturing [26,27,28,29].

Second, our findings that distress correlates with delayed initiation and non-use dovetail with clinic-based studies at the point of contraceptive start. Among adolescents initiating methods, depressive symptoms were associated with selecting less-effective methods and with ambivalence about pregnancy—an attitude that reliably predicts inconsistent use [30,31]. Francis and colleagues reported that mild depressive symptomatology tripled the odds of pregnancy ambivalence at initiation, suggesting that affective state can shape intention strength and follow-through even before side-effects or access hurdles enter the picture [31]. Together with our evidence that knowledge gaps (e.g., not knowing where to obtain contraception) interact with distress, these studies suggest a parsimonious mechanism: low mood increases decisional conflict and shortens planning horizons, which, when paired with misinformation or logistical barriers, is consistent with delays and non-use [30,31].

Third, the feasibility signals we saw in acute-care settings mirror and extend a growing ED literature. In a multi-center PECARN cohort, 15–17% of adolescent ED patients reported sex without contraception in the past year, with depressed females at particularly elevated risk—underscoring the ED as a high-yield venue for targeted prevention [32]. Trials and implementation work show that brief, point-of-care strategies can work: text messaging after ED discharge increased initiation in one randomized study, while a video-assisted, ED-based educational program improved LARC knowledge and interest in another [33,34]. Clinicians themselves view these workflows as acceptable, and adolescents endorse interest in receiving contraception or referrals during unscheduled care [35]. This literature supports our interpretation that same-day initiation models and “ED-first” counseling can translate the high interest we observed into concrete uptake, provided that protocols address confidentiality, insurance, and follow-up.

Moreover, the psychiatric inpatient signals (high desire for contraception information and initiation when access is facilitated) align closely with evidence from adolescent mental health units. In a Canadian psychiatric inpatient sample, most youth reported recent sexual activity, low contraception use, and substantial unmet informational needs, with many initiating care when offered on the unit [36]. U.S. provider surveys indicate favorable attitudes toward inpatient contraceptive provision but identify operational barriers (consent, device stocking, follow-up), echoing the access obstacles quantified in our included inpatient study [37]. Feasibility pilots of technology-supported sexual health education within psychiatric units further demonstrate acceptability and knowledge gains, suggesting a pragmatic path to embed standardized counseling alongside mental health care [38]. Collectively, these data support our conclusion that psychiatric admissions are a strategic “reachable moment” to pair stabilization with concrete reproductive health services.

These results must be interpreted in light of a bidirectional landscape in which mental health may both influence and be influenced by contraceptive experiences. Large registry studies have reported associations between hormonal contraception and subsequent antidepressant use or depression diagnoses—particularly among adolescents—while other population-based analyses and expert guidance caution about confounding and underscore wide inter-individual variability in mood responses to contraception [39,40,41]. Reciprocity is also plausible. Some adolescents with distress may have more healthcare touchpoints (e.g., mental health visits) that increase opportunities for contraceptive counseling or initiation, while others face motivational or logistical barriers that reduce uptake. Conversely, contraceptive side effects or fears may affect mood, with substantial inter-individual variability. These dynamics argue for tailored, symptom-sensitive counseling and careful wording that emphasizes associations rather than causation.

Based on observed associations, integrated models that pair mental health screening with contraception information and access may be promising, but require testing in designs that enroll adolescents with mental health conditions, include teen pregnancy endpoints, and address legal/ethical considerations for decision-making in acute settings. Collectively, the evidence suggests that adolescent reproductive health interventions must move beyond siloed models that separate mental health care from contraceptive counseling. Instead, an integrated framework—wherein mental health screening routinely triggers concurrent contraceptive needs assessment, and where high-access settings like EDs and psychiatric units are empowered to initiate or refer for contraception—could more effectively address overlapping vulnerabilities. Future research should focus on longitudinal, multi-setting interventions that examine not only uptake but sustained use, as well as the moderating effects of digital media exposure. Given the strength and consistency of associations observed, failure to address these intersecting domains risks perpetuating preventable adolescent pregnancies and the compounded health and social burdens they impose.

Generalizability beyond the United States is uncertain. In many LMICs, school-based education, community norms, and service availability differ substantially; linkages between distress, knowledge/access, and contraceptive use may therefore vary in form and magnitude. Dedicated reviews and context-specific studies are needed to assess similarities and identify distinct mediators.

Nevertheless, across the six included studies, only one reported a time-anchored association between the timing of first depressive episode and subsequent teenage pregnancy, indicating limited direct evidence. In contrast, multiple studies linked mental health symptoms to knowledge/access shortfalls and delayed or non-use of contraception—mechanisms that plausibly increase pregnancy risk. We therefore conceptualize two complementary pathways: a direct pathway (e.g., affective symptoms reducing planning/problem-solving at sexual debut) and an indirect/mediated pathway (symptoms → misinformation/knowledge gaps and access barriers → delayed initiation or non-use → higher pregnancy risk). Because available designs are observational and heterogeneous, these pathways should be interpreted as associational rather than causal. Future work should test mediation explicitly and prioritize teen pregnancy endpoints.

Given the paucity of eligible teen pregnancy endpoints, a follow-on review will prioritize longitudinal designs with pregnancy outcomes, include non-English records, and broaden geography beyond the U.S., to quantify direct effects and mediation by knowledge/access.

### 4.2. Limitations

This review has several limitations that should be considered when interpreting the findings. First, the heterogeneity of study designs, mental health measures, and contraceptive outcomes precluded quantitative meta-analysis, limiting our ability to generate pooled effect estimates. Mental health exposures ranged from validated multi-item scales (e.g., CES-D, DASS-21) to single-item self-reports, which may vary in sensitivity and specificity. Similarly, contraceptive outcomes were inconsistently defined, with some studies reporting self-reported non-use at last sex, others capturing delays in obtaining a method, and few documenting longitudinal adherence or discontinuation. We limited inclusion to English-language articles due to resource constraints for dual-review translation; we acknowledge the potential for language bias. Most included studies were cross-sectional, constraining causal inference, and even longitudinal designs were vulnerable to residual confounding from unmeasured factors such as relationship dynamics, intimate partner violence, and socioeconomic instability. All included studies were U.S.-based, potentially limiting generalizability to settings with different healthcare systems, cultural norms, and access infrastructures. Finally, reliance on self-reported behaviors introduces recall and social desirability biases, particularly in adolescent populations.

## 5. Conclusions

This review identifies associations—not causal effects—between adolescent mental health symptoms and contraception knowledge/access and use, with limited direct evidence for teen pregnancy. Knowledge and access gaps, especially among adolescents in psychiatric and acute care settings, emerge as actionable leverage points, with same-day initiation models in emergency departments showing promising feasibility and impact. In the context of pervasive social media misinformation, adolescents with mental health distress may be doubly disadvantaged, underscoring the need for integrated strategies that combine accurate contraceptive education, accessible initiation pathways, and mental health support. Embedding reproductive health services into mental health care encounters, enhancing digital health literacy, and leveraging high-contact care settings could collectively mitigate the intersecting risks identified in this review. These findings support a paradigm shift toward unified, cross-sector approaches to adolescent sexual and mental health promotion.

## Figures and Tables

**Table 1:** Risk of Bias Summary.

Study (Year)	Design and Appraisal Tool	Selection (NOS)	Comparability (NOS)	Outcome/Exposure (NOS)	Overall Judgment	Key Concerns/Notes
Harper et al., 2024 [23]	Repeat measures observational; NOS	★★★☆	★★	★★★	7/9 (moderate risk)	Selection of college sites; residual confounding despite adjusted mixed models
Vafai et al., 2020 [15]	National cohort (survival); NOS	★★★★	★★	★★★	8/9 (lower risk)	Strong design and timing; residual confounding possible but minimized
Casola et al., 2017 [24]	Cross-sectional (YRBS, weighted); NOS (adapted)	★★☆☆	★☆	★★☆	5/9 (moderate–higher risk)	Some outcomes bivariate only; adjusted ORs not reported in accessible sections
Berlan et al., 2025 [20]	Psychiatric inpatient cross-sectional; NOS (adapted)	★★★☆	★☆	★★★	6/9 (moderate risk)	Hospital-based sample; confounding by indication; strong outcome ascertainment
Hoehn et al., 2019 [19]	Pilot RCT; RoB 2	–	–	–	“Some concerns”	Randomization reporting and small sample size; low risk for deviations/missing data
Mollen et al., 2023 [25]	Implementation perspective; narrative only	–	–	–	Not appraised	Not synthesized; programmatic context only

Abbreviations: NOS, Newcastle–Ottawa Scale; RoB 2, Cochrane Risk of Bias 2; OR, odds ratio; YRBS, Youth Risk Behavior Survey; RCT, randomized controlled trial; Symbols: ★ = star awarded (criterion met), ☆ = no star (criterion not met); NOS domain maxima—Selection: 4, Comparability: 2, Outcome/Exposure: 3 (total max 9). For example, ★★★☆ = 3/4 in that domain.

**Table 2:** Characteristics of included studies.

Study (Year)	Country/Setting and Population	Design	n (Age)	Mental health Construct and Instrument	Contraception Knowledge/Access Variables	Outcomes Captured
Harper et al., 2024 [23]	USA; community colleges in TX and CA; students assigned female at birth	Repeated-measures observational (supplement to cluster RCT)	1665 (18–29; older adolescents and young adults)	Depressive symptoms (CES-D); anxiety and stress (DASS-21)	“Delayed getting the contraceptive method/prescription needed”	Delay in contraception (yes/no); multivariable aORs
Vafai et al., 2020 [15]	USA; nationally representative NCS-A; sexually active girls	Survival analysis (Cox)	1025 (13–18)	Timing of first depressive episode (CIDI-based DSM-IV)	NR	First teenage pregnancy (time from sexual debut); adjusted HRs
Casola et al., 2017 [24]	USA; Philadelphia YRBS; high school students (sexually active subset)	Cross-sectional, weighted	Weighted N = 29,755 (grades 9–12)	Past-year “sad or hopeless ≥ 2 weeks”, suicidal ideation, suicide attempt (single-item YRBS indicators)	NR	Contraception non-use at last sex; bivariate ORs (mental health, violence, substance use domains); multivariable model built (sex-specific)
Berlan et al., 2025 [20]	USA; psychiatric inpatient unit; adolescents assigned female at birth	Cross-sectional with EHR linkage	451 (14–17)	Psychiatric hospitalization context; sexual history	Did not know where to get contraception; difficulty getting to access	Interest in contraception information and in initiation during hospitalization; adjusted aORs
Hoehn et al., 2019 [19] *	USA; pediatric ED	Pilot randomized controlled trial	143 (adolescents)	NR	ED-based counseling/offer of same-day method	Same-day contraception initiation (ED visit); short-term uptake
Mollen et al., 2023 [25]	USA; pediatric EDs	Perspective/implementation overview (program model)	NR	NR	ED as service-delivery point	Narrative outcomes/feasibility points (no primary quantitative endpoints reported)

* Hoehn et al.’s (2019) [19] study was not restricted to mental health conditions; included as a downstream initiation outcome relevant to access linkages; Abbreviations: CES-D, Center for Epidemiologic Studies Depression Scale; DASS-21, Depression Anxiety Stress Scales–21; ED, emergency department; EHR, electronic health record; YRBS, Youth Risk Behavior Survey; NR, not reported; aOR, adjusted odds ratio; aHR, adjusted hazard ratio; OR, odds ratio.

**Table 3:** Associations between mental health indicators and contraception outcomes.

Study	Outcome (Definition)	Prevalence/Rate	Time Point/Note
Harper et al., 2024 [23]	Delayed contraception (needed method/prescription but delayed)	35%	Repeated surveys May 2020–Mar 2023 across 29 sites (TX, CA)
Vafai et al., 2020 [15]	First teenage pregnancy among sexually active girls	13% (unweighted n = 141/1025)	During observed risk time from sexual debut to interview/pregnancy
Casola et al., 2017 [24]	Contraception non-use at last sex (city-wide YRBS)	33% (weighted)	2015 Philadelphia YRBS; sexually active high-school students
Casola et al., 2017 [24]	Mental health indicators in sample	Sad/hopeless 34%; suicidal ideation 13%; suicide attempt 11%	Past-year indicators (single items)
Berlan et al., 2025 [20]	Interested in contraception information during admission	51.30%	Psychiatric inpatient survey + EHR linkage
Berlan et al., 2025 [20]	Interested in contraception initiation during admission	21.40%	Same as above
Hoehn et al., 2019 [19]	Same-day ED initiation of a method	26.8% (intervention) vs. 3.1% (control), *p* = 0.001	Index ED visit; randomized pilot (feasibility)
Mollen et al., 2023 [25]	NR (narrative/perspective)	NR	Programmatic overview of ED-based access

Abbreviations: ED, emergency department; NR, not reported; YRBS, Youth Risk Behavior Survey; *p*, *p*-value.

**Table 4:** Associations linking mental health and knowledge/access to contraception behaviors and teen pregnancy.

Study	Exposure (Predictor)	Outcome	Adjusted Effect (95% CI)
Harper et al., 2024 [23]	Depressive symptoms (CES-D)	Delayed contraception	aOR 1.58 (1.27–1.96)
Harper et al., 2024 [23]	Anxiety/stress (DASS-21)	Delayed contraception	aOR 1.46 (1.17–1.82)
Harper et al., 2024 [23]	Adolescents vs. young adults	Delayed contraception	aOR 1.32 (1.07–1.63)
Vafai et al., 2020 [15]	First depressive episode in same year as sexual debut	First teenage pregnancy	aHR 2.7 (1.15–6.34)
Casola et al., 2017 [24]	Suicidal ideation (past year)	Contraception non-use (last sex)	OR 1.93 (1.27–2.95)
Casola et al., 2017 [24]	Sad/hopeless ≥ 2 weeks	Contraception non-use	OR 1.78 (1.20–2.64)
Casola et al., 2017 [24]	Suicide attempt (past year)	Contraception non-use	OR 2.71 (1.62–4.54)
Berlan et al., 2025 [20]	Did not know where to get contraception	Interest in info	aOR 2.96 (1.60–5.50)
Berlan et al., 2025 [20]	Did not know where to get contraception	Interest in initiation	aOR 2.85 (1.43–5.68)
Berlan et al., 2025 [20]	Difficulty getting to access	Interest in info	aOR 3.33 (1.49–7.40)
Berlan et al., 2025 [20]	Difficulty getting to access	Interest in initiation	aOR 4.72 (2.03–10.95)
Hoehn et al., 2019 [19]	ED contraceptive program (vs. usual care)	Same-day initiation	26.8% vs. 3.1% (*p* = 0.001)
Mollen et al., 2023 [25]	NR	NR	NR

Abbreviations: aOR, adjusted odds ratio; aHR, adjusted hazard ratio; CI, confidence interval; ED, emergency department; OR, odds ratio; NR, not reported; Adjusted models per original reports; covariates summarized in Table 1.

## Data Availability

No new data were created or analyzed in this study. Data sharing is not applicable to this article.
